# Sleep staging through an unsupervised learning lens

**DOI:** 10.1016/j.patter.2025.101424

**Published:** 2025-12-12

**Authors:** Alexandros Christopoulos, Athina Tzovara

**Affiliations:** 1 Institute of Computer Science, University of Bern, Bern, Switzerland; 2 Center for Experimental Neurology and Sleep Wake Epilepsy Center-NeuroTec, Department of Neurology, Inselspital, Bern University Hospital, University of Bern, Bern, Switzerland

## Abstract

Sleep is one of the most essential parts of our daily lives. The gold standard for studying sleep is polysomnography (PSG) recordings. The first step of analyzing PSG recordings involves splitting them into sleep stages, which is performed manually. Machine learning algorithms have attempted to automate the tedious task of sleep scoring, mostly via supervised learning. A recent study in *Patterns* introduces AISleep, a novel algorithm approaching the task of sleep scoring in an unsupervised framework. This algorithm is based on humanly interpretable features and provides robust results across different datasets and age groups.

## Main text

Sleep is a landmark of healthy functioning, and it is commonly perturbed in a host of sleep-wake or psychiatric disorders. The gold standard for assessing sleep in healthy individuals and patients with sleep disturbances is polysomnography (PSG) recordings. These are routinely performed in sleep laboratories and allow sleep experts to study the composition and quality of sleep. A major bottleneck in the evaluation of PSG signals is that of sleep scoring or, in other words, splitting the recorded PSG data into sleep stages. This is highly dependent on manual labeling, which is time-consuming and has a moderate inter-rater agreement, ranging from 70%–80% depending on the stage of sleep.^1^ Multiple attempts have been made to create automatic sleep scoring algorithms in both the machine and deep learning domains, predominantly based on supervised learning.^1^^,^^2^ Unsupervised learning approaches for sleep staging remain, by comparison, sparser.^1^

A new study by Mai and colleagues presents AISleep, a novel unsupervised learning algorithm for sleep scoring that leverages the manifold patterns of PSG feature embeddings.^3^ The study starts by computing classical features of electroencephalography (EEG) signals based on the power spectral density of 30-s EEG epochs. The uniform manifold approximation and projection algorithm (UMAP) is then applied to these features to reduce their dimensionality in a 2D space. Individual sleep stages are identified sequentially into this space by computing a probability density function via a feature-weighted kernel density estimation. One of the strengths of this work is that while identifying each sleep stage, the study also explores the key EEG characteristics underlying each stage: wakefulness with eyes open, deep non-rapid eye movement (NREM) sleep, wakefulness with eyes closed, and then light NREM and rapid eye movement (REM) sleep.

Starting with wakefulness, the study identifies segments with eyes open. To achieve this, the distribution of gamma power is divided into low and high power in a data-driven way by employing a thresholding algorithm. Epochs with eyes open are identified from this procedure as those with high gamma power. The algorithm then moves on to identify deep NREM sleep (N2/N3). To do so, it splits the EEG signals into oscillatory and non-oscillatory (fractal) parts. Separating oscillatory and non-oscillatory EEG activity to study sleep follows a recent large body of literature.^4^^,^^5^ Eventually, personalized spindle power is extracted around 14 Hz, with consideration of variability in spindle activity. This allows the identification of N2/N3 epochs as those with a high spindle power.

To further differentiate between N2 and N3, the authors then detect slow oscillations based on an established algorithm^6^ and compute the percentages for each epoch. This is higher for N3 epochs, which allows them to be distinguished from N2 epochs. Next in line is the identification of wakefulness with eyes closed. This is assessed via the oscillatory EEG activity in the 5–20 Hz range, which, apart from spindles, also contains alpha activity, a hallmark of wakefulness with eyes closed. Finally, the remaining unlabeled stages are marked as presumed REM stages and eventually reattributed to N1 if needed, depending on their profile (duration or transition probability).

Although this work is based on unsupervised learning, the authors compute the resulting sleep scoring accuracy by comparing the sleep stages estimated by their algorithm with those of the manual scoring. Moreover, the accuracy of AISleep is compared with other benchmark algorithms, including a standard and an improved *k*-means algorithm and Gaussian mixture models. This comparison shows that AISleep achieves a high sleep scoring performance and significantly outperforms the remaining algorithms. An important addition to this comparison is an analysis assessing which sleep stages are more prone to mislabeling. The study reports that the majority of mislabeled epochs are N1 epochs, possibly due to the absence of specific features used by AISleep to identify those epochs or due to the relatively low number of N1 epochs over a night of sleep.

Remarkably, across benchmarks, AISleep matches or exceeds the performance of supervised algorithms. This key comparison is performed on new datasets that are not used to develop the AISleep algorithm. This validation represents an important step of ensuring that the results obtained in this study are not due to overfitting on a particular dataset but are capable of generalizing in unseen PSG recordings. The validation datasets used in this study are considered quite challenging, as they contain elderly participants, who often have alterations in their sleep characteristics,^7^ and patients with sleep disorders.

The study concludes with an interesting observation: all compared algorithms have a decrease in sleep scoring performance as a function of age. Authors report that among the studied EEG features, fast spindle power, the percentage of slow oscillations, and, more broadly, oscillatory EEG activity decrease with age. This observation could explain why distinguishing sleep stages in elderly individuals becomes more challenging and may even suggest that this particular task could require specialized algorithms in the future.

In summary, the study presented by Mai and colleagues^3^ employs unsupervised learning to address the challenge of automatic sleep staging. The study presents several advantages. First, it provides an explainable framework for sleep staging that relies on commonly used features of EEG signals such as slow waves and alpha, spindle, or gamma power. The study builds a stepwise algorithm for identifying sleep stages in a sequential manner. This approach could enable humans to trace algorithmic decisions reached when performing sleep staging. Second, this work employs three different datasets, both public and private, including healthy individuals and patients with sleep disorders, to develop and validate the sleep scoring algorithm, which shows that it can generalize to new contexts. Third, this work relies on a computationally accessible algorithm that provides robust results. Altogether, this study illustrates that complex and computationally expensive models, such as those relying on deep learning, are not necessarily required to perform the task of sleep staging. An open question for future work is how well these algorithms perform on additional sleep-wake disorders and demographic groups, which will determine their applicability in a clinical environment.

## Acknowledgments

A.C. is supported by the Swiss National Science Foundation (#320030_227728 to A.T.).

## Declaration of interests

The authors declare no competing interests.
